# Histologic, Histomorphometric, and Clinical Analysis of the Effects of Growth Factors in a Fibrin Network Used in Maxillary Sinus Augmentation

**DOI:** 10.3390/ijerph17061918

**Published:** 2020-03-15

**Authors:** Ihsan Caglar Cinar, Bahattin Alper Gultekin, Alper Saglanmak, Serdar Yalcin, Vakur Olgac, Eitan Mijiritsky

**Affiliations:** 1 Department of Oral Implantology, Istanbul University Faculty of Dentistry, Istanbul, 34093 Fatih, Turkey; cinarcaglar@gmail.com (I.C.C.); alper.saglanmak@istanbul.edu.tr (A.S.); profsyalcin@gmail.com (S.Y.); 2 Pathology and Oncology Cytology Department of Institute of Oncology, Istanbul University, 34093 Fatih, Turkey; volgac@istanbul.edu.tr; 3 Department of Otolaryngology, Head and Neck and Maxilllofacial Surgery, Tel-Aviv Sourasky Medical Center, Sackler Faculty of Medicine, Tel-Aviv University, 5219100 Ramat Aviv, Israel; mijiritsky@bezeqint.net

**Keywords:** beta-tricalcium phosphate, histomorphometry, maxillary sinus lifting, mineralized plasmatic matrix, volume change

## Abstract

This randomized controlled clinical trial evaluated the effect of mineralized plasmatic matrix (MPM), comprised of synthetic graft and platelet concentrates, on new bone formation and volume stability over time in maxillary sinus lifting (MSL). Unilateral MSL was performed in 20 patients with either beta-tricalcium phosphate (β-TCP) or MPM grafts (10 sinuses each). Six months postsurgery, specimens were obtained with a trephine bur prior to implant placement in 39 cases. Volumetric changes in sinus augmentation were analyzed between 1 week (T-I) and 6 months (T-II) postsurgery. Histomorphometric and histological analyses of biopsy samples revealed mean new bone percentages of 35.40% ± 9.09% and 26.92% ± 7.26% and residual graft particle areas of 23.13% ± 6.16% and 32.25% ± 8.48% in the MPM and β-TCP groups, respectively (*p* < 0.05). The mean soft-tissue areas in the MPM and β-TCP groups were 41.48% ± 8.41% and 40.83% ± 8.86%, respectively (*p* > 0.05). Graft reductions between baseline and 6-months postprocedure in the β-TCP and MPM groups were 17.12% ± 13.55% and 14.41% ± 12.87%, respectively, with significant graft volume reduction observed in both groups (*p* < 0.05) while there is no significant difference between MPM and β-TCP groups (*p* > 0.05). Thus, MPM, representing growth factors in a fibrin network, increases new bone formation and has acceptable volume stability in MSL procedures

## 1. Introduction

Tooth loss results in horizontal and vertical resorption of the residual bone; thus, maxillary sinus pneumatization can develop simultaneously after tooth extraction in the maxillary molar region [1,2]. To overcome this issue, various solutions have been proposed, such as the use of tilted implants applied in the anterior maxilla, short implants, zygoma implants and maxillary sinus augmentation combined with grafting procedures that implement autogenous bone, xenografts, allografts or alloplastic materials [3,4].

Boyne and James first reported using iliac bone as a grafting material for maxillary sinus floor augmentation [5]. Autogenous bone, with osteogenic, osteoinductive, and osteoconductive properties, is still considered to be the gold standard. [6,7]. However, grafting with autogenous bone is associated with donor site morbidity and extended duration of surgical procedures, and the volume of bone graft harvested may be insufficient for the requirements [8]. Biomaterials, thus, are promising substitutes for autogenous bone grafts in maxillary sinus augmentation. On the other hand, such biomaterials are degradable, non-antigenic, and biocompatible, known to stimulate osteogenesis and induce vascular growth [9,10]. Their major disadvantages are that they fail to exhibit ideal osteogenic and osteoinductive properties, despite having sufficient osteoconductive characteristics [11]. 

Consequently, biomaterials have recently begun to be used in hard tissue augmentation procedures, in conjunction with autogenous preparations, such as platelet concentrates and growth factors, to enhance their osteogenic properties in order to circumvent these problems [12]. Platelet-rich fibrin (PRF) is a second-generation platelet concentrate [13]. A fibrin network is gained by centrifugation of blood alone and comprises high amounts of platelets and leukocytes. The material obtained after centrifugation exhibits a natural autogenous material formed by non-proteolytic growth factors and fibrin tissue [14,15,16].

Platelet concentrates and beta-tricalcium phosphate (β-TCP) combination used in many medical fields. Szponder et al. [17] applied β-TCP and platelet-rich plasma (PRP) combination in the treatment of comminuted fractures in New Zealand white rabbits. After creating the tibial bone defects, β-TCP implants containing PRP were put into the fracture and the defect. After 12 weeks, the animals were killed and histological, radiological, peripheral quantitative computed tomography and scanning electron microscopy examinations were performed. They reported that β-TCP and PRP may be an effective method of bone union enhancement in the treatment of fractures in animals. Pascal Jungbluth et al. [18] stated that PRP on calcium phosphate bone substitutes promotes metaphyseal bone healing in mini-pigs. 

Mineralized plasmatic matrix (MPM), which is defined as a fibrin network that contains growth factors, is a bone graft material that contains a combination of beta-tricalcium phosphate (β-TCP) and PRF [19]. When a β-TCP and MPM mixture is prepared appropriately, the final product is easy to manipulate, and its unique structure may facilitate hard tissue augmentation. In addition to these mechanical properties that differentiate MPM from PRF, MPM also has advantages related to PRF, such as high levels of platelets and growth factors.

In many previous studies, various techniques for assessment of augmented bone graft volume have been reported [9]. 

The primary aim of this study was to evaluate the efficiency of MPM used in maxillary sinus augmentation for inducing new bone formation. We also compared β-TCP and MPM bone grafts in terms of volume changes at different time-points after sinus augmentation, by using CBCT scans. The null hypothesis of this study was that there would be a difference in new bone formation between the two groups. This article was organized according to the STROBE (Strengthening the Reporting of Observational Studies in Epidemiology) (http://www.strobe-statement.org) guidelines.

## 2. Materials and Methods

This study investigated partially edentulous patients who were scheduled for maxillary sinus augmentation at the Department of Oral Implantology, Istanbul University Faculty of Dentistry, from 2017 to 2018. The study was approved by the Ethics Committee of the Istanbul Aydın University Istanbul, Turkey, under protocol Number 2017-43. All participants provided written informed consent before the beginning of surgical procedures and the study was conducted according to the tenets of the Declaration of Helsinki.

The study was designed as a randomized controlled clinical trial. Participants were randomly allocated to the test group (MPM) and the control group (β-TCP). The inclusion criteria for this study were as follows: partially edentulous patients with a unilateral loss of teeth in the maxillary molar or premolar areas, with severe atrophy, and a residual alveolar ridge height of less than 5 mm. The exclusion criteria were as follows: patients with head and neck radiation therapy, uncontrolled diabetes, sinus pathology on radiographs, chemotherapy, and uncontrolled periodontal disease.

Twenty consecutive patients (8 women and 12 men), aged 38–66 years, were enrolled in this study. The cohort consisted of augmented maxillary sinuses analyzed with CBCT scans within 1 week (T1) of sinus elevation and after 6 months (T2), prior to implant placement.

### 2.1. Surgical Procedure

All necessary dental treatments were applied before the sinus lifting procedure. The sinus lifting operation was carried out under local anesthesia (Ultracain DS Forte, Sanofi Aventis, Istanbul, Turkey) in a staged surgical approach. The maxillary sinus was grafted utilizing a lateral window technique, as described by Kent and Block [20]. Chlorhexidine digluconate solution 0.12% was used to rinse patients’ mouths for 2 min prior to surgery. 

**Figure 1:**
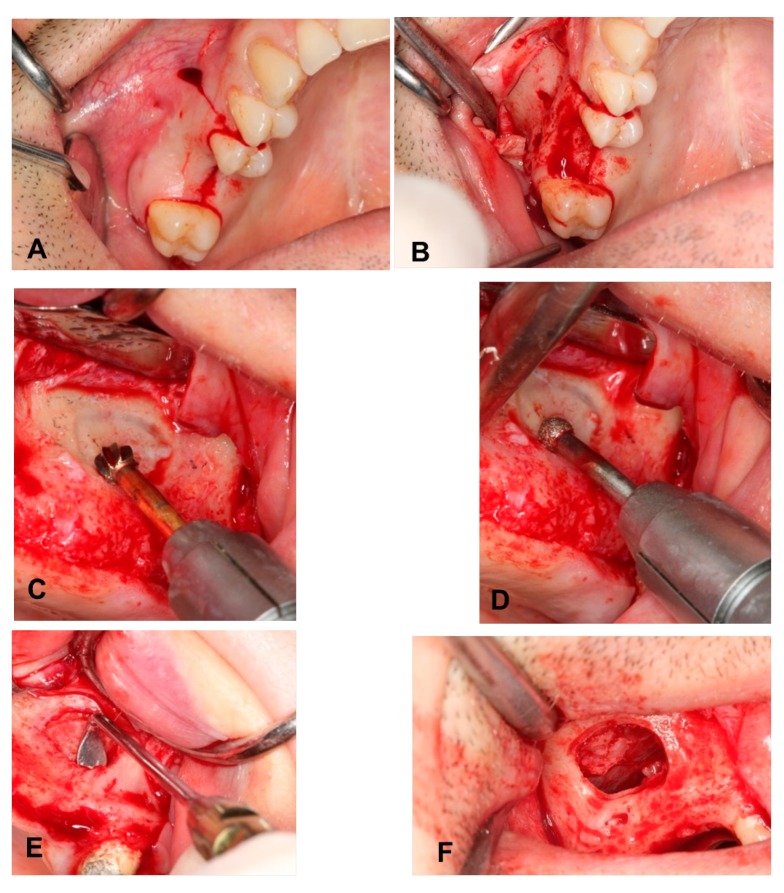
(**A**) Incision design. (**B**) Elevation of a muco-periosteal flap. (**C**) Preparing the lateral window with a steel bur. (**D**) Preparing the lateral window with a diamond bur. (**E**) Elevation of Schneiderian membrane. (**F**) Completely lifted Schneiderian membrane.

Briefly, via crestal and vertical incisions and muco-periosteal flap elevation, a lateral window was prepared with a dental carbide bur, followed by a wide-diameter diamond bur, to avoid sinus membrane perforation. The lateral window was dissected and reflected inward after the sinus mucosa was detached completely from the maxillary sinus walls (Figure 1).

### 2.2. Preparation of Biomaterials

**Figure 2:**
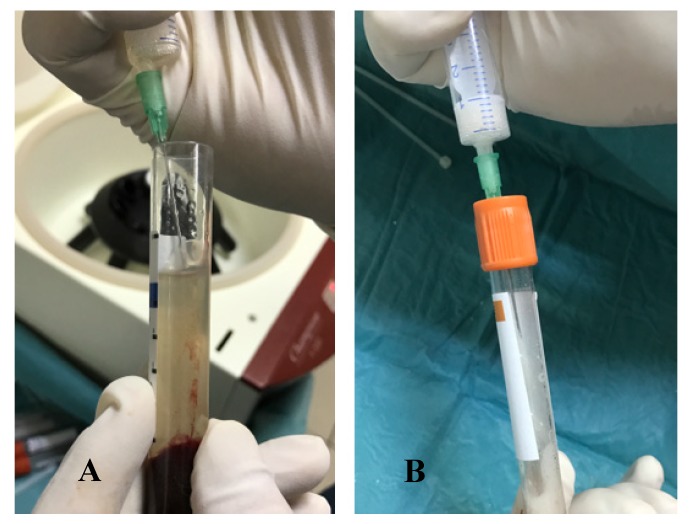
(**A**) The liquid in the mineralized plasmatic matrix (MPM) preparation tube was emptied into the container and mixed with beta-tricalcium phosphate graft (β-TCP) by a circular motion in one direction. (**B**) This process was continued until the MPM formed a unique structure.

**Figure 3:**
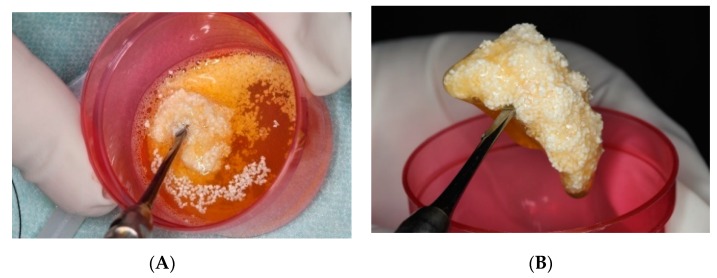
(**A**) The liquid in the mineralized plasmatic matrix (MPM) preparation tube was emptied into the container and mixed with beta-tricalcium phosphate graft (β-TCP) by a one-directional circular motion. (**B**) This process was continued until the MPM formed a unique structure.

**Figure 4:**
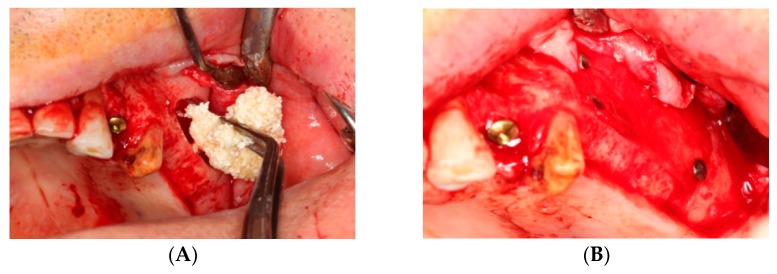
(**A**) Mineralized plasmatic matrix (MPM) material applied in maxillary sinus. (**B**) Grafted site was covered with a collagen membrane.

The MPM kit consisted of four 9-mL tubes without anticoagulant, one 9-mL preparation tube, two sterile containers, 0.5 cc β-TCP graft (Isios+, Atoll Implant, Toulouse, France), and a 6-mL injection syringe. Venous blood was drawn from each patient and the tubes were immediately centrifuged at 2700 rpm for 15 min (Ample Scientific Champion F-33D, Norcross, GA, USA), as recommended by the manufacturer. After centrifugation, any tube was selected and the upper one-third portion was aspirated with the aid of a syringe and discharged into the preparation tube (Figure 2). According to the manufacturer’s instructions, this tube was shaken five times. At this time, 0.5 cc of β-TCP was emptied into a sterile container and completely wetted with the upper one-third portion of the other blood tube. Subsequently, the liquid in the MPM preparation tube was emptied into this container and mixed with the β-TCP by a one-directional circular motion. This process was continued until the MPM formed a unique structure (Figure 3). For sinus floor augmentation, the MPM was packed into the maxillary sinus, and the grafted site was covered with a collagen membrane (Bio-Guide; Geistlich, Wolhusen, Switzerland) in the MPM group (Figure 4). The same steps were followed for patients in the control group, but the same β-TCP graft (Isios+, Atoll Implant, Toulouse, France) was used with saline wetting alone as the grafting material.

Postoperative care included antibiotic prophylaxis on the day of the surgery and for the following 7 days (1000 mg amoxicillin and clavulanic acid, Augmentin, twice daily), pain medication (600 mg ibuprofen, Apranax Fort, to be taken as needed, every 6 h), and 0.2% chlorhexidine mouthwash (Klorhex) twice daily for 2 weeks starting on the day after the operation. Dexamethasone (Kordexa, 4 mg daily) was administered for 2 days to minimize edema. The 4-0 nonabsorbable monofilament sutures (SERALON, Serag-Wiessner, Naila, Germany) were removed 10 days after surgery. Grafts were allowed to heal for 6 months before implant placement.

### 2.3. Histologic and Histomorphometric Analyses

After a healing period of 6 months, bone biopsy specimens were obtained from the augmented sites using a trephine bur with an external diameter of 3.0 mm (Helmut Zepf Medizintechnik GmbH, Seitingen-Oberflacht, Germany). The specimens were sent to the Pathology Institute at the University of Istanbul for processing and histomorphometric analysis. The specialist (VO) who performed this analysis was blinded to the grafting materials used. Bone biopsy specimens were fixed in 10% neutral buffered formalin for 48 h, decalcified in a mixture of 50% formic acid and 20% sodium citrate solution for 3 days, and then embedded in paraffin according to standard protocols. Blocks were stained with hematoxylin and eosin (H&E). Quantitative and qualitative analyses were performed using a light microscope (Olympus BX60; Olympus Corp., Lake Success, NY, USA) connected to a high-resolution video camera, interfaced to a computer running Olympus Analysis 5 histomorphometric software package. Percentages of new bone, residual graft particles, and fibrous or bone marrow tissue in the regions of interest were calculated.

### 2.4. Radiographic Analysis

**Figure 5:**
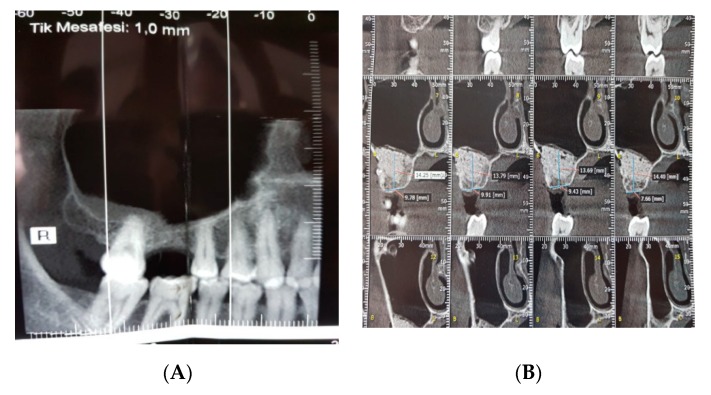
Preoperative (**A**) and postoperative (**B**) radiographic view.

**Figure 6:**
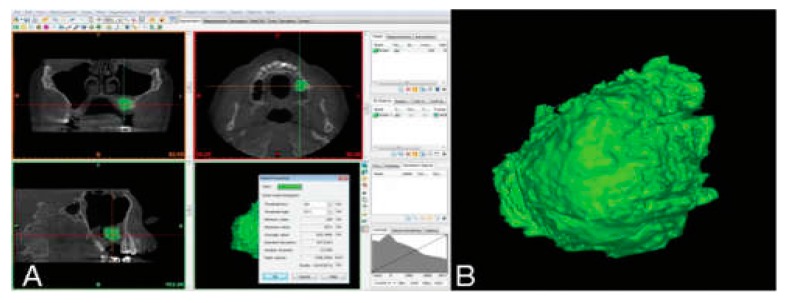
(**A**) The grafted volume was prepared manually with a threshold value consistent with the gray values of grafted and native bone, sinus cavity, and soft tissue expressed by the software program. (**B**) The augmented region was detached in varied colors and the volume of the graft was calculated in mm^3^.

Radiographic assessments were performed by CBCT to evaluate volumetric changes in graft sites (Figure 5). Images were acquired within 1 week (T1) as well as at 6 months after sinus augmentation (T2). Image analysis was performed with the i-CAT 3D imaging system (Imaging Sciences International, Hatfield, PA, USA). Imaging data of the augmented areas were sent to a new workstation, on which the volumetric changes in bone grafts were examined with MIMICS software (version 14.0; Materialise Europe, Leuven, Belgium) (Figure 6).

### 2.5. Statistical Analyses

A power analysis for the comparison of new bone formation between the MPM and β-TCP groups yielded the following results: power = 0.80, β = 0.20, and α = 0.05. Consistent with this estimation, the essential sample size was at least 10 subjects per group. The Number Cruncher Statistical System 2007 (Kaysville, UT, USA) was used for all statistical analyses. Descriptive statistical methods (minimum, maximum, mean, standard deviation) were used to evaluate the study data. The normal distribution of quantitative data was tested with the Shapiro–Wilk test and graphical investigations. While independent groups *t*-tests were used to compare quantitative variables between the two groups, the dependent groups *t*-test was used to evaluate the change observed over time within groups. The threshold of significance was set as *p* < 0.05.

## 3. Results

### 3.1. Clinical Observations

Twenty patients (8 women and 12 men; age range 38–66 years, mean age 53 years) who underwent 20 grafting procedures (*n* = 10 MPM and *n* = 10 β-TCP) completed the study. In four cases, Schneiderian membrane perforations occurred, but these were repaired and augmented simultaneously. All patients experienced uneventful healing without complications associated with the grafts. In total, 39 implants were placed into augmented areas. 

### 3.2. Histological and Histomorphometric Findings

**Figure 7:**
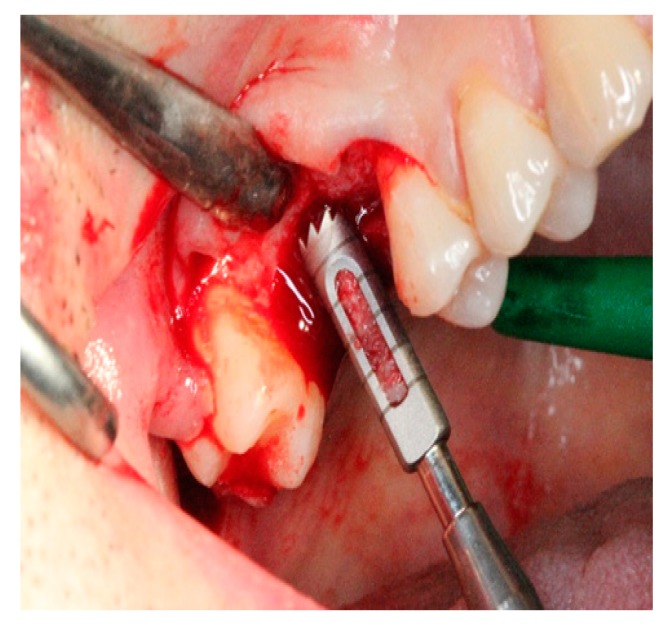
Biopsy specimens were collected with a trephine bur with an internal diameter of 3.0 mm.

**Figure 8:**
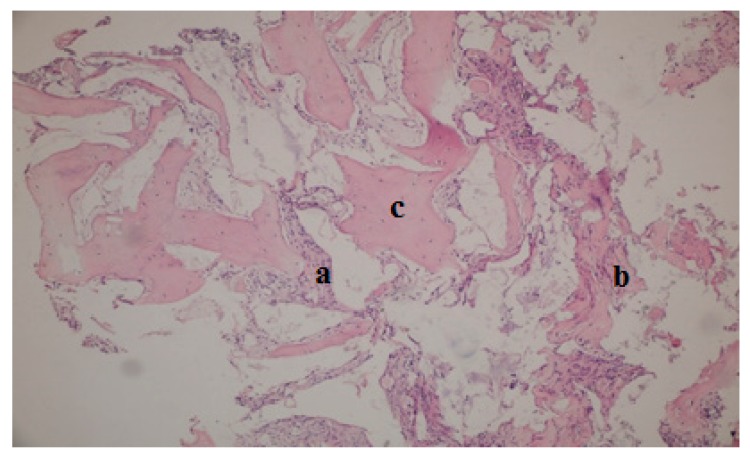
Light micrograph of a ground section of a specimen collected 6 months after maxillary sinus augmentation in the MPM group. (**a**) new bone, (**b**) residual graft and (**c**) connective tissue. (hematoxylin and eosin staining, ×100 magnification).

**Figure 9:**
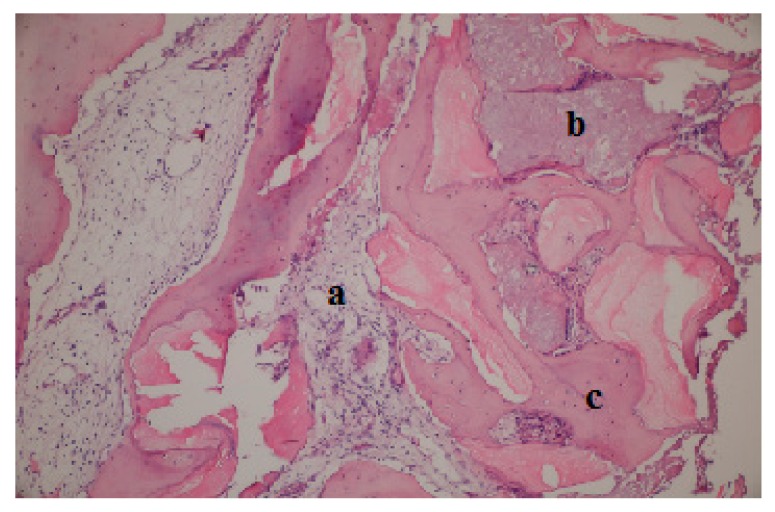
Light micrograph of a ground section of a specimen collected 6 months after maxillary sinus augmentation in the β-TCP group. (**a**) New bone, (**b**) residual graft, and (**c**) connective tissue. (hematoxylin and eosin staining, ×100 magnification).

Thirty-nine bone biopsy specimens were collected from the 20 patients during implant-bed preparations (*n* = 20 MPM group and *n* = 19 β-TCP group) and were subjected to histological and histomorphometric assessments (Figure 7). In the MPM group, new bone trabeculae and connective tissue in the form of thin fibrous bands were noted around the graft material, which was largely resorbed within the connective tissue in a loose structure (Figure 8). In some areas, an inflammatory cell infiltrate was observed around the graft particles or at the biomaterial–bone interface, but no signs of pathological inflammation were found. The residual graft particles seemed to be highly osteoconductive. In the β-TCP group, a loose structure of connective tissue around the residual graft material and new bone formation with apposition lines were observed (Figure 9). No inflammatory infiltrate was present at the interface with new bone or around the graft particles. 

**Figure 10:**
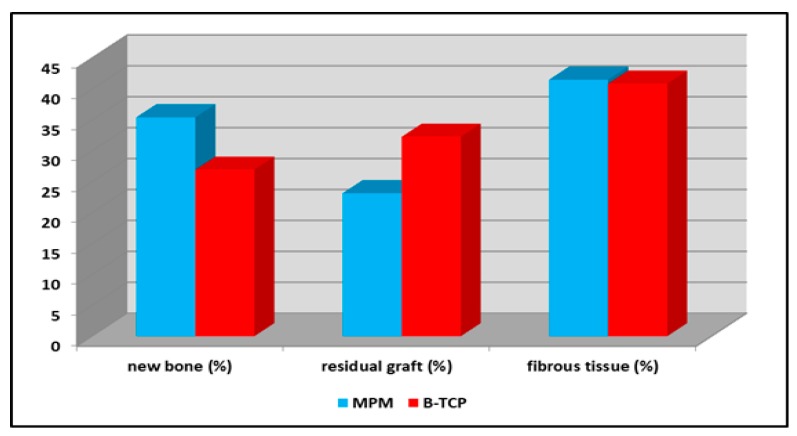
Mean percentages of the new bone, residual graft, and connective tissue between the MPM and β-TCP groups.

The mean percentages of new bone in the MPM and β-TCP groups were 35.40% ± 9.09% and 26.92% ± 7.26%, respectively (*p* < 0.05, Table 1). The percentage of residual graft particles was 23.13% ± 6.16% in the MPM group and 32.25% ± 8.48% in the β-TCP group (*p* < 0.05; Table 1). In addition, the percentage of fibrous or connective tissue was 41.80% ± 11.47% in the MPM group and 43.04% ± 13.76% in the β-TCP group (*p* > 0.05; Table 1 and Figure 10).

### 3.3. Radiographic Findings

Table 2 shows that, although there was no significant difference between the test and control groups’ graft volume at both T1 and T2 time-points, in patients receiving MPM and β-TCP, the average percent volume reductions were 14.41% and 17.12%, respectively, after 6 months of healing; this was a statistically significant difference (*p* < 0.01).

## 4. Discussion

In this study, we report the histologic, histomorphometric, clinical, and radiological results of using MPM, which comprises growth factors in a fibrin network. In this clinical study, we evaluated the new bone percentages between MPM and β-TCP groups after 6 months of healing and examined the volumetric changes in grafted sites after two-stage maxillary sinus lifting. There were significant differences in terms of percentages of new bone and residual graft particles between the groups, while the differences relating to fibrous tissue (soft tissue) areas were not significantly different between the groups. 

In the sinus lifting procedure, bone grafting substance selection is one of the critical factors that influence the final outcome [20]. This choice affects the increase in the vertical dimension of the bone crest and, therefore, the achievement of implant success and the long-term primary stability of the rehabilitation. Histologic and clinical outcomes regarding bone graft materials are still widely studied because an ideal bone graft material should ensure volume maintenance, provide biologic stability, and induce a high rate of new bone formation and bone remodeling [21,22]. Donor site morbidity and limited availability are disadvantages of using autogenous bone grafts, although bone substitutes are associated with virus transmission and are immunogenic, which remain major causes for concern. Another disadvantage of autogenous bone is its unpredictable and high resorption rate [23,24]. 

Because of these negative properties, several grafting materials, such as synthetic calcium phosphate bone grafts and polymers, may be alternative materials for maxillary sinus lifting. Several prospective controlled examinations have been reported to date, but only a few randomized clinical trials have addressed the histologic and histomorphometric analysis of bone samples after sinus lifting, particularly as related to the use of MPM to date. MPM serves as a platelet concentrate and contains growth factors; it is thus similar to PRF and also functions as a bone substrate that can form a unique structure with β-TCP.

In the present study, in the MPM and β-TCP groups, 35.40% and 26.92% new bone formation were found after a healing period of 6 months, respectively, raising the possibility that MPM may induce new bone formation in sinus lifting procedures when used as a bone graft. El Moheb et al. [25] performed an animal study and concluded that MPM was effective in inducing bone formation. MPM and β-TCP were applied into surgically created defects in sheep. Biopsies were obtained at 2 and 8 weeks after surgery for histological examination. The MPM group showed more woven bone formation and was more stable at 2 weeks. As a result, the authors reported that MPM should be considered as an alternative to bone graft in guided bone regeneration procedures. 

Wiltfang et al. [26] compared the use of β-TCP alone with that of β-TCP plus PRP in sinus lifting and reported an average bone formation of 29% in the β-TCP alone group and 38% in the β-TCP plus PRP group. Kim et al. [27] compared β-TCP, recombinant human bone morphogenetic protein 2 (rhBMP-2)-coated β-TCP, and PRF mixed with β-TCP in their efficacy on bone formation in sinus lifting on rabbits. Histologic specimens were obtained at 3 days, 1 week, 2 weeks, 4 weeks, 6 weeks, and 8 weeks. At all time-points, the β-TCP group showed the least new bone formation. New bone formation percentages were found to be significantly higher in the PRF mixed with β-TCP group than in the rhBMP-2-coated β-TCP group. 

In the literature, there are some controversial studies related with PRF when applied in sinus lifting as a graft material alone or combined with other bone graft substances. Liu et al. [28] aimed to execute a meta-analysis to evaluate the efficacy of PRF in maxillary sinus lifting procedure. Embase, Pubmed and Cochrane Library were searched and only randomized controlled studies were identified. There were no statistical differences in new bone formation, survival rate, percentage of residual bone graft, contact between bone substitute and newly formed bone and soft-tissue area between the non-PRF and PRF groups. Nizam et al. [29] performed 26 maxillary sinus augmentation procedures using deproteinized bovine bone mineral (DBBM) and leukocyte- and platelet-rich fibrin (L-PRF) mixture in test group or deproteinized bovine bone mineral alone in control group. Bone samples were harvested from the implant sites 6 months postoperatively for histomorphometric and histological analyses. They stated that the addition of L-PRF in DBBM did not enhance the amount of the graft integrated into the newly formed bone or the amount of regenerated bone under histomorphometric and histological evaluation. In our study, MPM group showed higher new bone formation rather than β-TCP group. This result could be related with our centrifuge technique, application form, RPM variety or machine’s *g* force. 

Suba et al. [30] reported similar new bone ratios of 34.7% in an autogenous bone control group and 32.4% in a β-TCP group. Zijderveld et al. [31] reported a clinical study comparing maxillary sinus lifting using autogenous bone and β-TCP. They reported new bone ratios of 17.0% in the β-TCP group and 41.0% in the autogenous bone group, which indicated that the autogenous bone graft was more efficient. 

In our study, the percentage of remaining particles was higher with β-TCP (32.25%) than with MPM (23.13%). Nevertheless, β-TCP showed some signs of resorption that were consistent with an experimental study that investigated β-TCP in extraction sockets of beagle dogs [32]. In that study, β-TCP was applied into sockets with PRP or alone. At 12 weeks after surgery, early resorption of the graft particles was noted. New bone was organized both in the expanded pore system of the granules and along their boundaries. Satisfactory bone density in the grafted site was gained through integration of the granules into the newly organized bone by 24 weeks. 

Yilmaz et al. [33] reported the effect of β-TCP and PRF alone or in combination on bone formation on tibial defects in pigs. They applied β-TCP, PRF, and a β-TCP + PRF combination in critical-sized defects and examined the specimens histologically after 12 weeks. The combination PRF + β-TCP yielded the highest amount of newly formed bone, but the difference from other groups was insignificant. Bolukbasi et al. [34] used PRF mixed with β-TCP in sheep’s tibial defects to determine the efficacy of PRF in inducing bone regeneration. They revealed that the use of PRF with a β-TCP bone graft increased the amount of bone formation and that the efficacy of PRF depended on the co-used biomaterial, as well as on its own properties. These results should be considered with caution, as animal experiments may differ markedly from those involving humans.

The association of platelets and β-TCP was also used in other medical fields, for example in aesthetic medicine for improving the soft tissues. Sambhav et al. [35] reported that β-tricalcium phosphate and platelet rich fibrin combination with coronally advanced flap have been shown to be a successful and promising approach for the treatment of furcation defect. Its gaining clinical attachment importantly manages both the furcation involvement and gingival recession simultaneously. Tozum TF et al. [36] treated periimplantitis defect with β-tricalcium phosphate and platelet rich fibrin combination and they stated that this combination showed improved early wound healing and might influence acceptable regeneration. 

Del Fabbro et al. [37] reviewed 24 trials researching whether autologous platelet concentrates were beneficial for treatment of periodontal disease and concluded that PRP may supply benefits when applied in combination with bone graft materials for the treatment of intrabony defects. Taschieri et al. [38] stated that the in immediate postextraction implant placement study, P-PRP group showed better wound healing and soft tissue management in the first 7 days.

Choukroun et al. [39] investigated the use of freeze-dried bone allograft in conjunction with PRF to improve bone regeneration in a sinus lift procedure. The results presented notable reduction in healing time in other words, healing period was reduced by half from 8 to 4 months. Combination of PRF and bone graft can lead to the volume reduction of the bone substitute, and this seems to promote revascularization of this graft by improving angiogenesis.

From the results of our clinical study, it can be concluded that the resorption rates of MPM and β-TCP are approximately 14.41% and 17.12%, respectively, at the 6-months’ follow-up. When we compared our resorption-rate scores with those of other studies, such as those using synthetic bone substitutes or autogenous bone as graft material [40], our results supported those of other studies. 

Various techniques have been proposed to measure volumetric changes of bone graft materials [41]. Panoramic radiographs display 2D images and therefore do not allow measurement of volumetric changes in graft materials. Recently, CBCT has gained more popularity, as it provides easy usage and lower cost, and X-ray compared with conventional CT can therefore be used in a wider range of patients. 

We analyzed a 3D dataset and volumetric changes in bone grafts by means of MIMICS software and found that 3D data acquisition improved the information on the grafted sites. Volumetric data taken by CBCT have high measurement validity and a relative bias below 1% with respect to the same measurements accomplished during operation. However, choosing the region of interest in separate CBCT sections remains prone to error. The MIMICS software used in our study inserted the spotted areas between different images, and thereby increased the reliability of our data. Gultekin et al. [42] analyzed cone beam computed tomography scans of sinus lift procedure grafted with mineralized allograft (MA), deproteinized bovine bone (DBB), or a mixture of MA and DBB as a composite and measured to evaluate the volume of the augmented sinus. The average percent volume reductions were 19.38% ± 9.22%, 8.14% ± 3.76%, and 24.66% ± 4.68% for MA, DBB, and composite graft, respectively. MPM and β-TCP showed similar resorption rate with MA; deproteinized bovine bone could offer greater volume stability during healing period. Ohe et al. [43] applied biphasic calcium phosphate combined with PRF in maxillary sinus lifting procedure as a graft material and investigated volume stability over the time by 3D CT analyzing software program. Six months after surgery, average volume reduction was 15.68%. 

Smolka et al. [44] reported that average percent volume reduction of autogenous bone was 16% at 6 months’ follow up and 19% at 12 months’ follow-up in maxillary sinus lifting. Johansson et al. [23] reported 49.5% volume reduction in maxillary sinus augmentation with autogenous graft material. Autogenous bone has unpredictable resorption rate in hard tissue augmentation as a graft material. 

Umanjec-Korac et al. [41] reported 21% volume reduction in maxillary sinus lifting with xenograft material in 29 patients at 2-year follow-up. Sbordone et al. [45] applied autogenous bone as a graft material in maxillary sinus augmentation and reported 39.2% volume reduction at 6-year follow-up. The shared feature of all these studies exhibiting higher resorption rates than our study was that their re-entry/observation periods are longer compared to our 6 months. It is known that graft materials placed in the sinus resorb slowly over time.

In this study we applied the trapezoidal flap. This flap design allows the clinicians to visualize operation area which is very important if there is any complication during surgery for example sinus membrane perforation. It would be easier to repair complications with this flap design in sinus lifting procedure. The modified triangular flap design has been designed in the literature but trapezoidal flap design is superior to manage potential complications in maxillary sinus lifting surgery and has been used by many investigators in the literature [23,42]. 

The use of barrier membranes in terms of facilitating treatment success and implant survival is highly controversial. Many authors reported no differences in implant success between barrier membrane covered and uncovered groups [46]. Other studies reported higher implant survival rates when bone defects were covered with a membrane [47]. Previous studies showed markedly lower amounts of soft tissue formation in maxillary sinuses covered with barrier membranes than in those without such a covering [48]. Covering the bone defect with a barrier membrane can improve osteoconductive properties, avoid soft tissue invasion, and support the immobility of the bone graft, which is very important for the ossification process. Thus, we used a collagen membrane to cover the augmented areas both in control and test groups.

The volumetric stability of the bone substitutes that were used in the sinus lifting procedure can represent an important issue for implant success, which can be achieved by having a satisfactory bone quality as well as quantity [49]. 

In the present study, we obtained biopsy samples, vertically, through the alveolar crest. Zijderveld et al. [31] performed lateral biopsies, whereas Szabó et al. [9] and Suba et al. [30] performed vertical biopsies from the alveolar crest. Bone formation within the maxillary sinus proceeds evenly from the periphery of the bone graft towards its center. This suggests that the area near the maxillary sinus window is quite unsuitable for assessing bone formation. Thus, lateral biopsies may not exactly reflect osseointegration in the area surrounding the implant. Also, for ethical reasons, to avoid second wound site creation, we preferred to harvest bone specimens vertically with a 3.0-mm-diameter trephine bur after a healing period of 6 months.

The number of available biopsies for both the control and test groups in our study were comparable to that in most of the previous published articles [18,20,26]. It should be noted that, since harvesting of the bone specimen and implant bed preparation were accomplished with a trephine bur, the biopsy removal was limited by the need to preserve suitable dimensions at the implant site to ensure primary stability when placing the implant; for implants of 10 mm in length, the sites had to be prepared appropriately. In some cases, consequently, the residual sinus floor dimensions suggest that the trephine bur may not have extended sufficiently deeply into the bone graft to obtain an adequate biopsy sample. It might be considered that, if drilling with the trephine bur was accomplished as apically as possible, a smaller amount of bone specimen would have remained attached to the grafted portion of the maxillary sinus. Thus, for the safety of the procedure, it was advisable not to infringe the sinus floor during implant insertion. 

In present study, we gathered bone specimens for histologic and histomorphometric analyses at 6 months after the sinus augmentation procedure. Various studies have revealed a relationship between vital bone formation and graft maturation time. Tosta et al. [50] treated maxillary sinuses with β-TCP at 9 months and reported a higher amount of new bone formation than in our study. Froum et al. [51] reported that they achieved the highest amount of new bone formation in a β-TCP graft that was harvested 8 months after maxillary sinus lifting. The histomorphometric outcomes of our study showed that the amount of vital bone could be increased by a longer healing time; this compares favorably with the reported literature [9,38]. Additional clinical studies with greater sample sizes are necessary to determine the efficacy of healing time on the amount of bone formation and whether longer healing times are needed with β-TCP- and MPM-grafted sinuses.

We designed the present study to include patients with residual bone height of the maxilla of <5 mm. We applied two-stage sinus lifting with an external approach technique to improve our ability to compare results between the test and control groups. When planning the protocol of this clinical study, it was considered important to include patients with alveolar ridge dimensions within a distinctly defined range. This was considered important for assessing the differences between the two bone graft materials, which will be the subject of a different report.

Residual alveolar ridge dimensions in other studies of sinus lifting may not necessarily be revealed in this way. In some studies, this may introduce an element of bias into the examination, as it may be difficult to determine whether implant success, for example, is associated with the residual alveolar bone of the posterior maxilla or with the support offered by the new bone formed after augmentation. In our study, we compared two groups with comparable alveolar ridge dimensions who were treated with similar surgical protocols, except for the bone graft materials used. This is particularly significant, since the amount of residual graft and new bone may be dependent on the distance between the residual bone and the grafted area.

The inclusion criteria for this study were partially edentulous patients with a unilateral loss of teeth in the maxillary molar or premolar areas with severe atrophy and a residual alveolar ridge height of less than 5 mm. Both MPM and β-TCP showed clinically satisfactory results, but in the literature, there are many studies such as osteotome sinus floor elevation technique [52] and osseodensification [53] when residual bone height is limited in maxillary posterior region. Further investigations were needed for extreme atrophic cases (for example 0–1 mm residual height) whether MPM could be successful. We gathered bone biopsies for histologic and histomorphometric analyses at 6 months after the sinus augmentation procedure. MPM consists growth factors and could accelerate the bone formation. As time was a limitation for our study, MPM could provide clinically acceptable results over a shorter period (for example 3–4 months after surgery). 

In our study, we applied β-TCP bone graft with MPM in the test group and β-TCP bone graft solely in the control group. The MPM kit consisted of four 9-mL tubes without anticoagulant, one 9-mL preparation tube, two sterile containers, 0.5 cc β-TCP bone graft, and a 6-mL injection syringe. Growth factors are susceptible to β-TCP granules and show satisfactory effects on tissue regeneration [54,55]. Xenogeneic bone graft materials resorb slowly and growth factors are not affinitive to xenografts. 

## 5. Conclusions

Within the limits of this clinical study, our results proved that growth factors in a fibrin network, MPM, could increase new bone formation significantly, as compared with β-TCP. MPM, being biocompatible and easy to manipulate, is crucial in bone-regeneration procedures. Nevertheless, the number of related studies in the current literature is limited. Further clinical and experimental studies are required to confirm the efficacy of MPM for bone formation in guided bone regeneration, particularly in comparison to autogenous bone grafts that are still considered to be the gold standard in augmentation procedures.

## Figures and Tables

**Table 1:** The percentages of new bone, residual graft, and fibrous tissue in test and control groups.

Title	MPM (*n* = 20)	β-TCP (*n* = 19)	*p*
**New bone (%)**	35.40 ± 9.09	26.92 ± 7.26	0.003 **
**Residual graft (%)**	23.13 ± 6.16	32.25 ± 8.48	<0.001 **
**Fibrous tissue (%)**	41.48 ± 8.41	40.83 ± 8.86	0.817

Independent groups *t*-test. ** *p* < 0.01.

**Table 2:** Bone graft volumes of both MPM and β-TCP groups.

Volume (mm^3^)	MPM (n = 20)	β-TCP (n = 19)	^a^ *p*
Post-op 1 week	2622.78 ± 521.92	2670.12 ± 546.85	0.845
Post-op	2256.78 ± 664.15	2240.03 ± 680.85	0.956
Difference (%)	14.41 ± 12.87	17.12 ± 13.55	0.675
^b^ *p*	0.017*	0.002*	

^a^ Independent group t-test; ^b^ Dependent group t-test * *p* < 0.05.
